# Biological Variation of Corrected QT and QRS Electrocardiogram Intervals: Interpreting Results of Drug-induced Prolongation

**DOI:** 10.5811/westjem.33602

**Published:** 2025-07-12

**Authors:** Alan Wu, Kayla Kendric, Caitlin Roake, Emily Kelly

**Affiliations:** * University of California, San Francisco, Department of Lab Medicine, San Francisco, California; † University of California, San Francisco, Department of Emergency Medicine, San Francisco, California; ‡ University of California, San Francisco - Fresno, Department of Emergency Medicine, Fresno, California

## Abstract

**Introduction:**

Toxicologists use a universal threshold to determine QRS and QTc prolongation in poisoned patients. Further understanding of the biologic variance of these intervals may allow for a more personalized approach to assessing the clinical significance of electrocardiogram (ECG) changes in these patients.

**Methods:**

We recruited six male and six female healthy subjects. Standard 12-lead ECGs were performed in duplicate once per week for four consecutive weeks. We calculated the mean and standard deviation, the coefficient of variance (CV) for replicate readings (CV_A_), and within (CV_I_) and between individuals (CV_G_) using analysis of variance for all subjects and separately for males and females. From these measured parameters, we determined the index of individuality (II), the reference change value (RCV), and number of readings needed to maintain a homeostatic setpoint.

**Results:**

The median QRS interval for healthy males (103.4 milliseconds [ms]) was statistically higher than that for females (88.6 ms) in our study (*P* < .05). The CV_A_ and CV_I_ for the QRS interval for the total cohort were relatively low at 3.0 and 2.2, respectively. The CV_G_ for the QRS interval was relatively high at 12.9. There was no difference in the QTcorrected (QTc) interval between gender (404 vs 415 msec, respectively). The II was 0.29 for QRS and 0.74 for QTc in pooled subjects. The RCV was 10.3 and 7.1 msec, respectively, for QRS and QTc for all subjects. The number of samples needed to establish a homeostatic set point was 1 for all analyses at a closeness of 10% with a 95% probability (*P* = .05).

**Conclusion:**

We demonstrated a significant difference in QRS duration between healthy males and females as well as a low II, particularly for the QRS interval, indicating that the CV_G_ is greater than the CV_I_ among these ECG intervals. In this study we also determined that one ECG is needed to establish a homeostatic set point for patients. If a baseline ECG is available, medical toxicologists would benefit from using the baseline tracing as an internal reference for determining QRS and QTc prolongation in the individual patient rather than a predetermined universal threshold for managing poisoned patients.

## INTRODUCTION

Biologic variation refers to the concept that there is variation in biological attributes between and within the individual over time and in different physiological states.^1^ Within a single subject, a source of the variation comes from fluctuations around an average value. Variation may also arise from pre-analytical factors (ie, whether an individual is standing or sitting, well hydrated or not, their fasting status). Additional sources of variation are introduced in sample collection, handling, and the analysis itself. Biologic variation becomes important when analyzing values in a medical setting, as clinicians must determine whether a given data point is deviated from the norm because of a disease state or whether this is part of normal variability for a given individual.

One outcome of analyzing the biological variation of laboratory and clinical data has been the discovery that for certain analytes there is a high degree of variation between individuals, while for others there is relative stability among individuals. As a result, certain data is best interpreted by comparing an individual to a population of patients, while others are better interpreted by comparing that individual to his/her/their self at a baseline health status. Understanding these nuances can support the goal of making medicine a more personalized and targeted.

Within the realm of laboratory medicine, the primary literature is replete with medical applications of biological variation data. Of relevance to emergency medicine practices are the calculations for high- sensitivity cardiac troponin.^2^ This marker has a low index of individuality; therefore, serial measurements are more relevant than use of a population-based reference intervals.^3^

In overdose and poisoning, the electrocardiogram (ECG) is a rapid tool for interrogating the degree of ion channel blockade at the cardiac level.^4^ This can aid in determining the source of the overdose, guiding treatment, and assessing the efficacy of the treatments delivered. Measured ECG intervals are calculated automatically, comparable between sequential ECGs, and are relatively straightforward to interpret. Analysis of ECG intervals in cases of suspected poisoning can identify patients likely to have serious cardiac events.^5^ One study found that the QRS duration was more accurate in predicting adverse impacts such as seizures from tricyclic antidepressant overdose than serum drug concentrations.^6^

The duration of the QRS interval is determined by sodium ion influx in cardiac myocytes.^4^ Many medications and toxins can cause blockade of voltage-gated sodium channels and prolongation of the QRS interval including anticholinergic medications, tricyclic antidepressants, and anti-dysrhythmics.^4^ In determining the level of sodium channel dysfunction, a cutoff of QRS duration of 120 milliseconds (ms) has been used classically, although some research studies suggest cutoffs as low as 100 ms.^7^

The duration of the QT interval, which is corrected by the heart rate to give the QTcorrected (QTc), is determined by efflux through potassium channels during the depolarization phase of the cardiac cycle. Medication classes that affect the QTc are numerous and include antipsychotics, the fluoroquinolone antibiotics, and the macrolide antibiotics. A QTc duration (using the Bazett formula^8^) of greater than 450 in males and 470 in females has been proposed as a cutoff for medically significant prolongation; different cutoffs are proposed for alternative rate-correction formulas.^9^

**Population Health Research Capsule**

What do we already know about this issue?

*The QTc and QRS intervals are provided with each 12-lead ECG. When prolonged, these values inform emergency physicians that a patient may be suffering from a toxic side effect of a drug*.

What was the research question?

*We determined the QT and QRS intervals in healthy subjects to determine the biological variability of these measures*.

What was the major finding of the study?

*Rather than using a fixed cutoff that defines prolongation of the QTc interval from an ECG, optimum use of this parameter would be to establish a within-individual cutpoint*.

How does this improve population health?

*A fixed cutoff is used today to determine an abnormal result for the QTc and QRS interval. Using a personalized medicine approach will improve the accuracy of drug toxicity*.

From previous studies, we know that there is significant biological variation in ECG intervals. Other research groups have found that the QTc shows high intersubject variability, and additionally that the formula which best corrected the QT interval for rate differed between individuals.^10^,^11^ Previous studies have also found substantial differences in the QRS duration between males and females.^12^

While prior studies have addressed biologic variation in ECG intervals, none to date have established an approach to apply this variation to proposing a cutoff that can guide clinical management for an individual patient. In this study we investigated the variability of the QRS interval in healthy individuals to establish the degree of inter-individual and intra-individual variability in these measurements. We argue that the high degree of inter-individual variation and low degree of intra-individual variation in these intervals necessitates a more personalized approach to ECG interpretation in the poisoned patient, and we suggest methods to accomplish this.

## METHODS

### Subjects

We recruited six male (mean age 39±16 y range 27–70 years) and six female healthy subjects (mean age 37±9 y range 30–48 years, *P*>0.05 in age between genders). Due to the small numbers of subjects enrolled, we did not attempt to age match the participant’s gender. Through self-disclosure, each subject denied a history of coronary artery disease, diabetes, hypertension, heart failure, or structural electrographic abnormalities. Laboratory testing was not conducted to verify the medical history. Each subject signed a written informed consent per study protocol. Standard 12-lead electrocardiograms (ECG) were performed in duplicate (Model CP150, Welch Allyn, Skaneateles Falls, NY) once per week for four consecutive weeks. For correction of the QT interval to heart rate, this instrument uses the Bazett formula, the one that is the most frequently used. The leads were removed and repositioned before each replicate ECG reading. This study was reviewed and approved by the Institutional Review Board of the University of California, San Francisco and Western Institutional Review Board.

#### Statistical Analysis

We used an analysis of variance (ANOVA) for calculation of the summary statistics with both sexes and separately (MedCalc, Inc., ver. 19.6.4, Ostend, Belgium). The Shapiro-Wilk test was used to determine normality. We calculated mean coefficient of variance for replicate readings (CV_A_), within (CV_I_) and between individuals (CV_G_) using an ANOVA for all subjects and separately for males and females. We applied the Reed criterion to determine whether any outliers were statistically significant warranted rejection.^13^ From these measured parameters, we determined the index of individuality (II), reference change value, and number of readings needed to maintain a homeostatic setpoint using established formulas.^2^ All ECG intervals were checked manually by a physician to exclude machine computational failure.

## RESULTS

The Figure shows the raw QRS and QTc results for all subjects and the Table shows the mean/median results for males, females, and combined sexes. (There were no outliers. Both the automated interpretation provided by the ECG instrument and a manual review of the tracing showed that all ECGs were without defect, including none with a bundle branch block. The QRS results for females were parametrically distributed; however, for males, they were not and, therefore, median results are reported. The QTc results were parametrically distributed for males and females and the combined group and, therefore, mean results are reported. The Table also shows the calculated values for CV_A_, CV_I_, CV_G_, II, reference change value, and number of samples needed to establish a homeostatic setpoint.

Consistent with previous reports, the median QRS interval for healthy males was statistically higher than that for females even with a small sample size (100 and 88, respectively, *P* < 0.05).^10^ The CV_A_ and CV_I_ for the combined group were low at 3.0% and 2.2%, respectively. The CV_G_ was higher at 12.9, resulting in an index of 0.29 (0.27 for males and 0.83 for females). An index of less than 0.6 indicates that a population-based reference interval is of no value.^1^ Therefore, this test is most useful for monitoring serial change. The RCV, which takes into consideration both the imprecision and the measurement itself as well as the biological variation, was 10.3% for the combined group (9.2% for males and 11.7% for females).

There was no difference in the mean QTc interval by gender (404 milliseconds [ms] vs 415 msec, respectively). The CV_A_ and CV_I_ for the combined group were also low at 1.9% and 1.6%, respectively. The index of individuality was 0.74 when pooling all subjects (0.60 for males and 1.39 for females). The RCV was 7.1% for the combined group (7.6% for males and 6.4% for females). The number of samples needed to establish a homeostatic set point for both the QRS and QT_C_ was one for all analyses at a closeness of 10% (*P* = .05).

## DISCUSSION

The ECG is a crucial diagnostic instrument for medical toxicologists in the work up of poisonings and overdoses. Various foreign substances, known as xenobiotics, prolong the QRS and QTc intervals, and associated ECG findings are used to predict clinically significant toxicity and help guide management in poisoned patients. This study, which included 12 healthy individuals, demonstrates a low index of individuality for these intervals, particularly for the QRS interval, indicating that inter-individual variability in QRS duration is greater than that of intra-individual variability. A statistically significant difference in QRS duration between males and females was also observed. Consequently, variability must be considered when interpreting ECG results in clinical toxicology to avoid misdiagnosis and ensure accurate assessment of cardiac toxicity in cases of poisoning and overdose.

Many xenobiotics are known to prolong the QRS and/or QT intervals, and these findings are often interpreted as objective signs of toxicity in patients with suspected overdoses. For instance, tricyclic antidepressants (TCA) are well documented as prolonging the QRS interval by blocking the fast voltage-gated sodium channels in the myocardium, leading to prolonged ventricular depolarization. In a prospective analysis of ECGs in TCA-poisoned patients, the maximal limb lead QRS duration was found to be prognostic of seizures and ventricular dysrhythmias. Specifically, the study demonstrated that the risk of seizures was 0% if the QRS duration was less than 100 ms and 30% if it was greater. Similarly, the risk of ventricular dysrhythmias was 0% if the QRS duration was less than 160 ms and 50% if it was greater. We concluded that the QRS duration is more accurate in predicting adverse outcomes than serum TCA concentrations.^6^

As a result of these findings, many medical toxicologists consider a QRS duration of 120 ms or longer, although some propose lower cutoff such as 100 ms.^4^ This coupled with other ECG findings such as a prolonged terminal R wave have shown to be a reliable predictor of serious cardiovascular and neurological toxicity in TCA overdose, which may prompt earlier alkalinization such as with 1–2 milliequivalents per kilogram bolus of sodium bicarbonate, and longer periods of observation.^14^

Beyond drug-induced QRS prolongation, there are several other physiological conditions known to cause prolonged QRS intervals. For instance, patients with left ventricular hypertrophy exhibit a longer QRS duration because the greater mass of the left ventricle generates most of the heart’s electrical forces. Similarly, a bundle-branch block results in a prolonged QRS interval as the ventricles depolarize sequentially rather than concurrently. While bundle-branch blocks can occur spontaneously, they may also signal toxicity, particularly due to the effects of fast sodium-channel blocking agents.^15^ These agents include amantadine, bupropion, carbamazepine, cocaine, cyclic antidepressants, diphenhydramine, lamotrigine, phenothiazines, quinidine, and other type IA and IC antidysrhythmic medications. Increased QRS complex duration is also observed in patients with hypothermia, hypermagnesemia, and hyperkalemia. Recognizing these various causes is essential for accurate diagnosis and treatment in both clinical and toxicological settings.

The QT interval, measured from the beginning of the QRS complex to the end of the T wave, exhibits normal biological diurnal variation and is influenced by factors such as autonomic tone, age, gender, the method of acquiring the ECG, and observer variability.^16^,^17^ The QT interval also varies with heart rate, being prolonged at slower heart rates and shortened at higher heart rates. Due to this variation, multiple formulas have been developed to calculate a corrected QT interval (QTc), which estimates the QT interval corrected to a standard heart rate of 60 beats per minute (bpm). The Bazett formula is the most commonly used method and provides an accurate QTc for heart rates between 60–100 bpm.

According to the Bazett formula, the QTc is considered prolonged if it exceeds 450 ms in males and 460 ms in females.^8^ However, the Bazett formula tends to overcorrect at heart rates above 100 bpm, leading to inaccurately prolonged QTc values. Some medications, such as bupropion, that are thought to prolong the QT interval may result in a “prolonged” QTc due to the increased heart rate caused by the xenobiotic.^18^ To address this limitation, other formulas that more accurately calculate QTc at high heart rates have been developed, although it remains uncertain which formula is optimal.^19^ A QT nomogram that plots QT interval duration against heart rate may better predict the risk for lethal dysrhythmia.^20^ A QTc interval greater than 500 ms correlates weakly with an increased risk of developing ventricular dysrhythmia, and this threshold is often used by medical toxicologists to monitor and manage toxicity from medications known to prolong the QTc.^21^,^22^ Others have suggested the half-the-reference-range rule for determining QT prolongation.^23^ None of these are statistically valid approaches.

The findings from this study can be used to justify altering clinical practices within the context of medications that can prolong these intervals. Rather than use pre-established cutoffs to indicate drug toxicity, it would be best practice to first measure baseline QTc and QRS intervals prior to the initiation of treatment. Then repeat ECG measurements can be taken as part of therapeutic monitoring. An increase beyond the reference change value (RCV) of the marker would indicate a statistically significant increase. However, it is insufficient to just use the RCV values obtained from the biological variation studies conducted in health. It may be necessary to find a higher difference from baseline to indicate or predict complications due to the drug, warranting a change in therapy in terms of drug or dose. For subjects seen in the emergency department, previous ECG readings are typically available from previous visits, a routine practice that is conducted today for patients suspected of acute coronary syndromes.

## LIMITATIONS

This study has several limitations. First, it only included healthy individuals, which is usually the first step in such studies.^1^ However, results do not account for how biological variation might be altered in patients on a therapeutic or overdose of relevant medications. This will be the subject of our subsequent research studies. Second, this study does not address how an elevated heart rate due to xenobiotic toxicity may alter the QT interval, nor does it provide guidance on the best method to calculate the QTc in these scenarios. Overall, while the findings support the use of individualized ECG interpretation by referencing an individual’s baseline intervals as reference standards for possible interval prolongation, further research is needed to understand how poisoning and elevated heart rates due to toxins affect ECG intervals and to refine the approach for calculating QTc in these situations.

This study is also limited by the small number of enrollments, as most clinical trials report on a higher number of participants. However, biological variability studies are usually conducted on recruitments of between 10–20 subjects. This is because previous studies have shown that while increasing the number of enrollments reduces the 95% confidence interval, it does not substantially alter the estimates for CV_A_, CV_I_, CV_G_ or the calculated parameters (index of individuality, reference change value and homeostatic set point, and a smaller number of samples helps reduce the pre-analytical variables.^1^ To further justify the small sample size used in this study, two original landmark biological variation studies using limited enrollments were conducted on serum creatinine (n=15),^24^ and high sensitivity cardiac troponin (n=12),^2^ both markers demonstrating a low index of individuality. These reports led to the adoption of the estimated glomerular filtration that includes age, sex, and muscle mass to reduce inter-individual variability, and the need for serial testing for the early rule out of acute coronary syndromes, respectively.

## CONCLUSION

The biological variation of ECG intervals demonstrated in this study, as evidenced by low reference change values along with known physiological conditions affecting these intervals, suggests that medical toxicologists should consider an individualized approach when determining QRS and QTc prolongation if a baseline ECG is available. Rather than relying on a universal threshold, the approach of comparison to prior individual baseline ECG intervals could allow for more accurate assessment of drug-induced cardiac effects in poisoned patients. The use of an individual’s baseline ECG, as justified by the biological variation data presented in this study, may be superior to using a pre-established cutoff, or obviate the need for optimizing QTc formulas or creating nomograms.

While having a truly asymptomatic baseline ECG for which to refer is ideal, there are challenges. Today, ECGs are typically only obtained when there is medical need to do so; therefore, an abnormal tracing may invalidate this approach. Therefore, to adopt this practice, baseline ECGs would be required during health, which adds to healthcare costs and difficulties in retrieving such baseline results. We also conclude that only one ECG is necessary to establish a homeostatic set point, or reference baseline, for patients. This implies that if a baseline ECG is available, the coefficient of variance within individuals (CV_I_) could be applied to the baseline QRS and QTc intervals to identify any drug-induced changes.

## Figures and Tables

**Figure:**
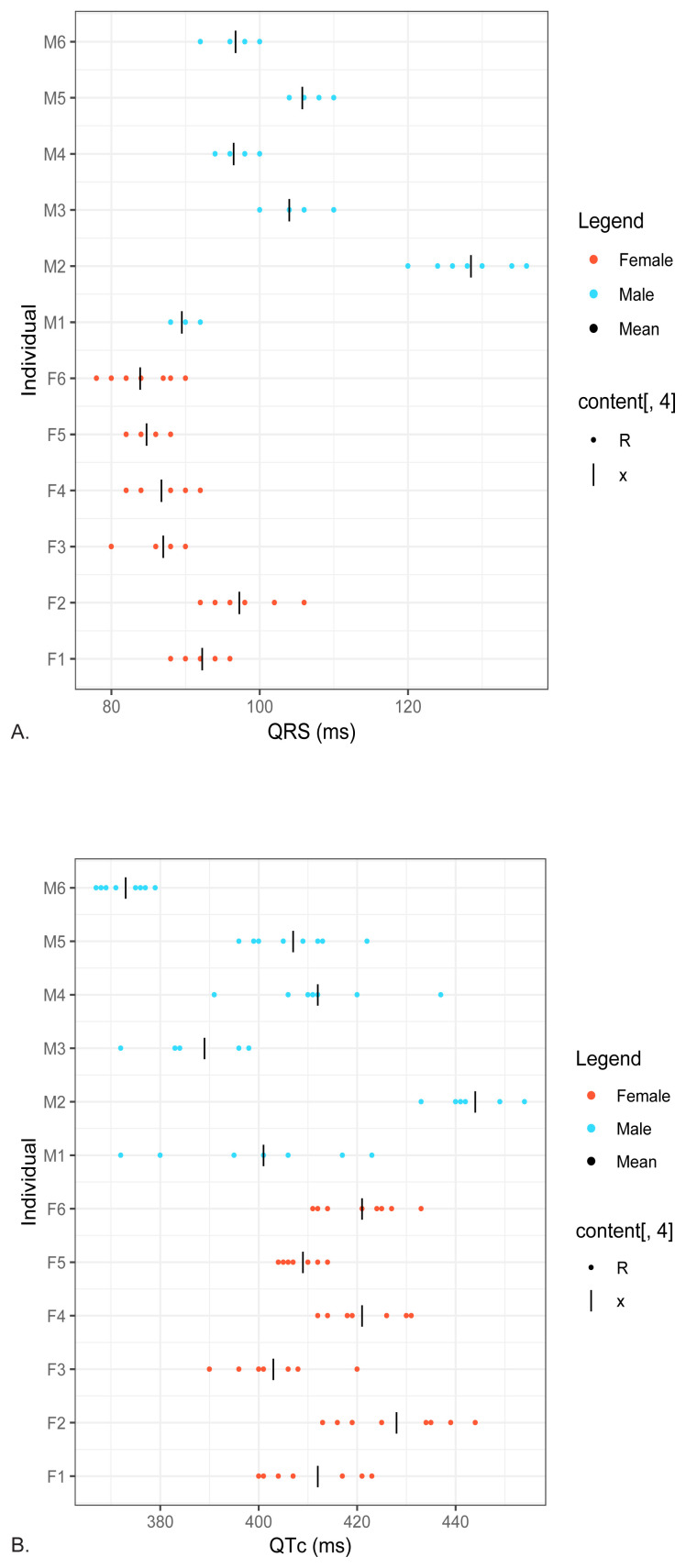
The figure shows the raw results for all subjects, and the Table summarizes the results of this study. A. QRS interval. B. QTc interval. Red dot females, blue dots males, and the black line represents the mean of both sexes. Each dot may represent more than one reading. *ms*, millisecond.

**Table:** Biological variation of the QTc and QRS intervals from the electrocardiogram.

Parameter	QRS			QTc		
	
	Males	Females^a^	Total	Males	Females^b^	Total
Mean (ms)				404.2	415.5	410
Median (ms)	100.0	88.0	94.0			
Standard deviation (ms)	10.2	5.1	14.2	4.0	9.3	13.8
CV_A_	2.5	3.7	3.0	2.5	1.2	1.9
CV_I_	2.2	2.0	2.2	1.1	2.0	1.6
CV_G_	12.4	5.1	12.9	4.6	1.7	3.4
Index of individuality	0.27	0.83	0.29	0.60	1.39	0.74
Reference change value	9.2	11.7	10.3	7.6	6.4	7.1
Samples for homeostatic setpoint	1	1	1	1	1	1

a P < 0.05 males vs. females for median values.

b P > 0.05 males vs. females for mean values.

*ms*, millisecond*s*; *CV_A_*_,_ coefficient of variation between readings; *CV_I_*, coefficient of variation within individuals; *CV_G_*, coefficient of variation between individuals.
